# High-grade focal areas in low-grade central osteosarcoma: high-grade or still low-grade osteosarcoma?

**DOI:** 10.1186/s13569-015-0038-7

**Published:** 2015-10-29

**Authors:** Alberto Righi, Anna Paioli, Angelo Paolo Dei Tos, Marco Gambarotti, Emanuela Palmerini, Manuela Cesari, Emanuela Marchesi, Davide Maria Donati, Piero Picci, Stefano Ferrari

**Affiliations:** Department of Pathology, Rizzoli Institute, Via di Barbiano 1/10, 40136 Bologna, Italy; Department of Oncology, Rizzoli Institute, Bologna, Italy; Department of Orthopaedic Oncology, Rizzoli Institute, Bologna, Italy; Department of Pathology, Treviso Regional Hospital, Treviso, Italy

**Keywords:** Low-grade osteosarcoma, Central, High-grade, Chemotherapy, Prognosis

## Abstract

**Background:**

High-grade foci (grade 3 according to Broder’s grading system) are sometimes detected in low-grade (grade 1 and 2) central osteosarcoma. The aim of this study was to retrospectively evaluate the clinical outcome in patients upgraded to high grade (grade 3) after a first diagnosis of low-grade osteosarcoma, following the detection of high-grade areas (grade 3) in the resected specimen.

**Methods:**

Of the 132 patients with a diagnosis of low-grade central osteosarcoma at surgical biopsy at our Institute, 33 patients were considered eligible for the study.

**Results:**

Median age was 37 (range 13–58 years). Location was in an extremity in 29 patients (88 %). Post-operative chemotherapy was given in 22 (67 %) patients. Follow-up data were available for all patients, with a median observation time of 115 months (range 4–322 months). After histological revision, areas of high-grade (grade 3) osteosarcoma accounting for less than 50 % of the tumor were found in 20 (61 %) patients, whereas the majority of the tumor was composed of a high-grade (grade 3) component in 13 (39 %) patients. In the 20 cases of low-grade osteosarcoma with high-grade foci (grade 3) in less than 50 % of the tumor, 9 patients did not receive adjuvant chemotherapy; only one of them died, of unrelated causes. In the adjuvant chemotherapy group (11 out of 20 patients), one patient developed multiple lung metastases and died of disease 39 months after the first diagnosis. In the other 13 cases of low-grade osteosarcoma with high-grade foci (grade 3) in more than 50 % of the tumor, 12 patients received adjuvant chemotherapy: 2 had recurrence, 4 developed multiple lung metastases and 3 died of disease. The only patient who did not receive chemotherapy is alive without disease 232 months after complete surgical remission.

**Conclusion:**

Our data indicate that patients with a diagnosis of low-grade osteosarcoma where the high-grade (grade 3) component is lower than 50 % of the resected specimen, may not require chemotherapy, achieving high survival rates by means of complete surgical resection only.

## Background

The grading of malignant bone tumors has traditionally been based on a combination of histologic diagnosis and Broder’s grading system, which assesses cellularity and degree of anaplasia [1]. The 7th edition of the AJCC Cancer Staging Manual recommends a 4 grade system, with grades 1 and 2 being considered “low-grade” and grades 3 and 4 “high-grade”. The World Health Organization endorses the use of a two-tier system designating an osteosarcoma as low-grade (grades 1 and 2 in a four-tier system) or high-grade (grades 3 and 4 in a four-tier system) [2].

Histologic grading of osteosarcoma has an important impact on clinical outcome: the risk of distant metastases is low in grade 1–2 (low-grade) and high in cases of grade 3–4 (high-grade) osteosarcoma, making chemotherapy mandatory in patients with a diagnosis of grade 3–4 (high-grade) osteosarcoma [3, 4].

Low-grade central osteosarcoma is an uncommon variant, accounting for approximately 1–2 % of all osteosarcomas composed of low-grade, mostly fibroblastic, osteogenic proliferation featuring mild cytological atypia [5, 6]. The low-grade central osteosarcoma series published in the literature report an incidence of areas of high-grade osteosarcoma ranging between 10 and 36 % of cases [3–5, 7–12]. Some authors regard the high-grade component of low-grade central osteosarcoma as a separate entity from conventional high-grade osteosarcoma, and consider it a form of morphologic progression (dedifferentiation) of a low-grade osteosarcoma [7–9, 11, 12]. While this distinction is controversial and not uniformly accepted, it appears that low-grade tumors with focal high-grade progression actually behave differently than their conventional high-grade counterparts [7–9, 11]. Unfortunately there are no established criteria (such as the percentage of the overall tumor with a high-grade component) to categorize these tumors as typical high-grade (grade 3–4) osteosarcoma [3–5]. In principle, the presence of any high-grade area in a low-grade lesion makes the tumor high-grade, thereby prompting systemic adjuvant treatment [13–15]. Nevertheless, there is little data in support of such therapeutic approach, and it is not well established whether low-grade (grade 1–2) central osteosarcoma with areas of high-grade (grade 3) osteosarcoma actually differs from high-grade (grade 3–4) osteosarcoma, with regard to rates of local recurrence, metastasis, and survival.

The aim of this study was to retrospectively evaluate the clinical outcome in patients who, after a first diagnosis of low-grade central osteosarcoma based on surgical biopsy, were upgraded to high-grade (grade 3) osteosarcoma following evaluation of the surgical specimen at resection.

## Methods

**Fig. 1:**
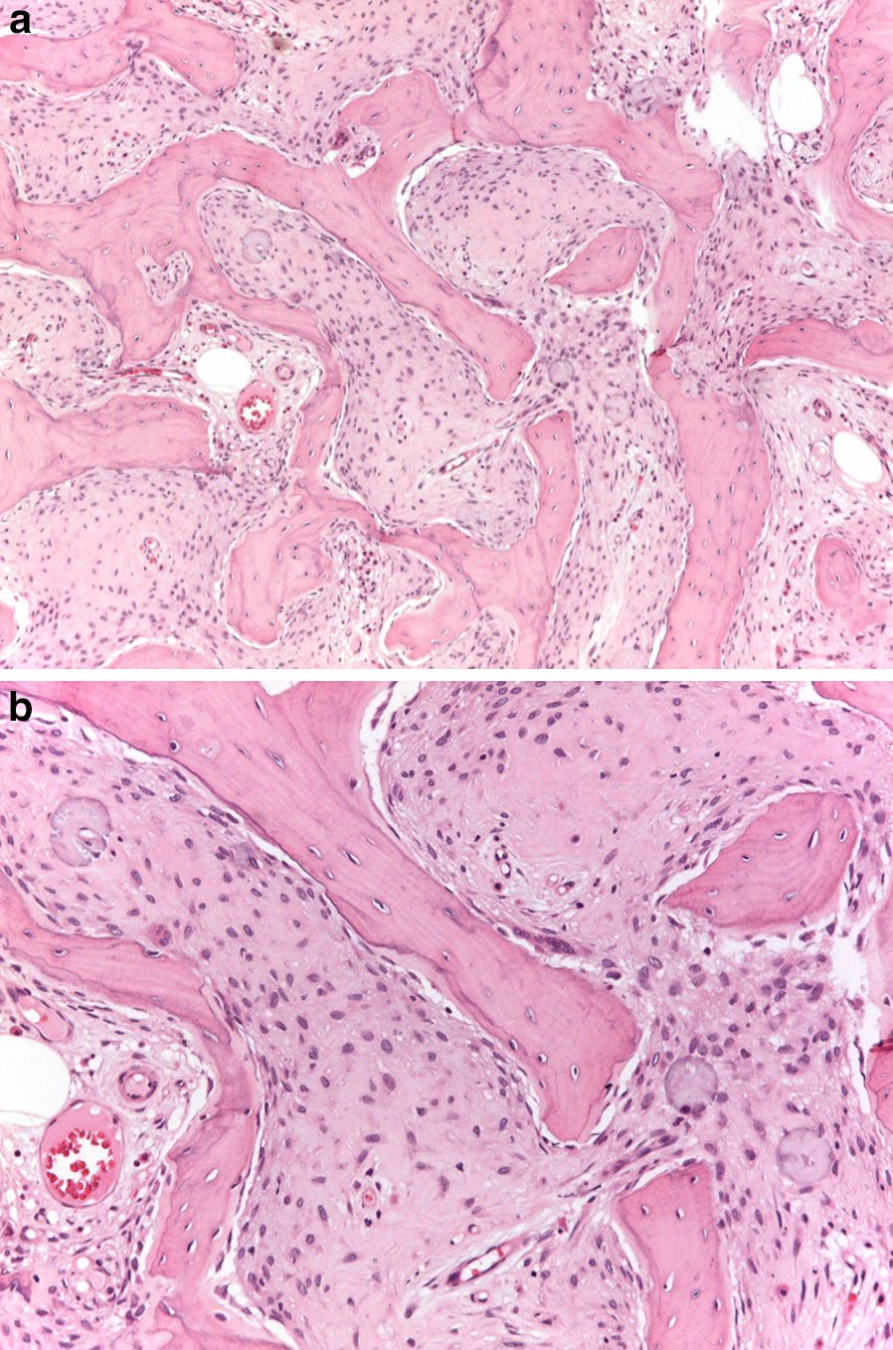
**a**, **b** A low grade (grade 2) fibroblastic osteosarcoma, fibrous dysplasia-like variant (case no. 18)

**Fig. 2:**
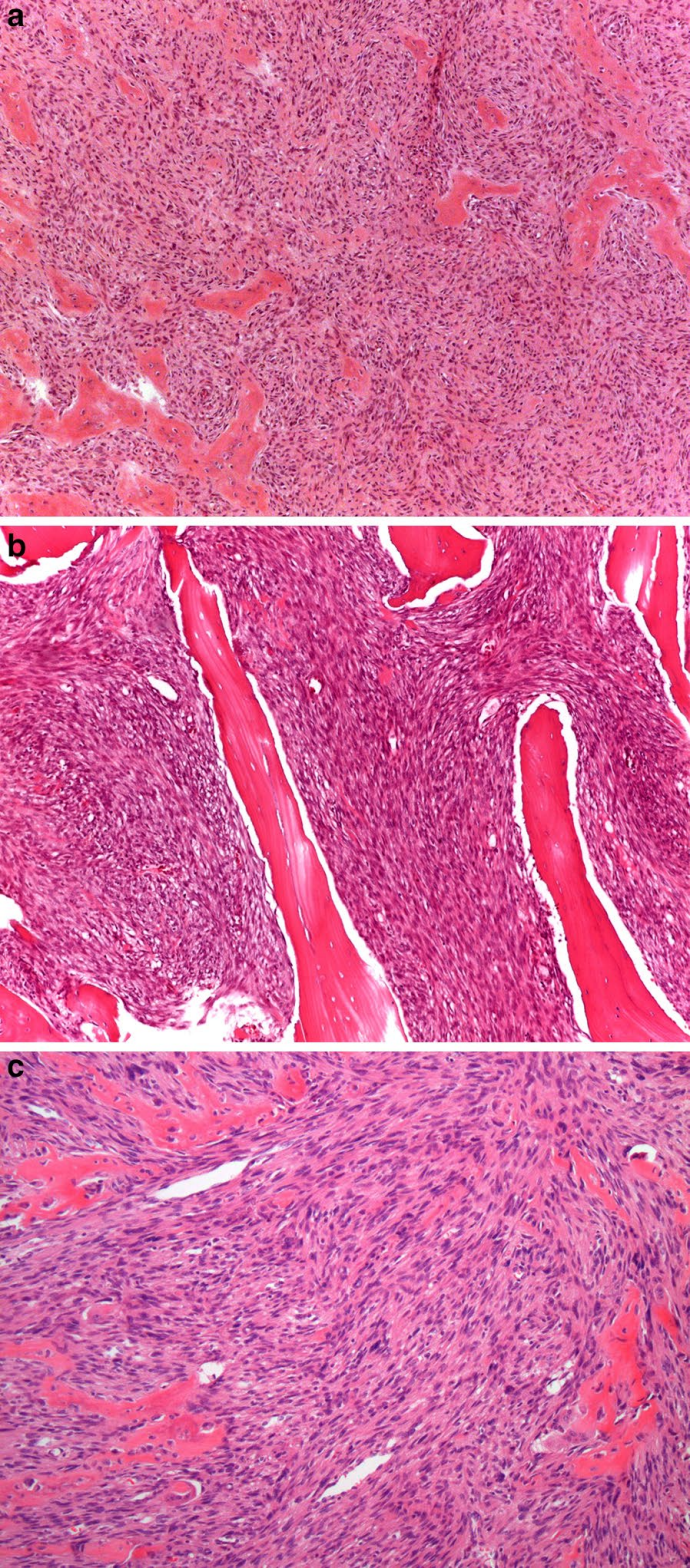
**a**–**c** An example of a case with high-grade (grade 3) areas characterized by the presence of increased cellularity, lack of typical architectural pattern of growth of low grade osteosarcoma and higher nuclear atypia

The medical records were retrieved of a consecutive series of patients diagnosed and treated for low-grade osteosarcoma (grade 1–2 according to Broder’s grading system) at the Rizzoli Institute, Bologna, Italy, between January 1981 and June 2014 (Fig. 1a, b). The resected specimens and radiological imaging were reviewed. No patients received preoperative chemotherapy. A statement on consent to use the data for scientific purposes was obtained from all patients. Inclusion criteria were: (a) availability of the histologic slides of both the biopsy and surgical specimens, in which systematic mapping of the entire tumor featured a low-grade osteosarcoma according to the protocol for the examination of bone tumor specimens [16]. Three pathologists (A.R., M.G., A.D.T.) independently reviewed the slides stained with hematoxylin and eosin, evaluating diagnosis, subtype, and grade. On the basis of the predominant morphology of the neoplastic cells and quality of the intercellular matrix, osteosarcomas were classified in surgical specimens by the following subtypes: osteoblastic, chondroblastic and fibroblastic. Tumours were graded according to the 4-tiered Broder’s grading system by assessing cellularity and degree of atypia [1]. Where high-grade (grade 3) areas were detected (defined as the presence of increased cellularity, absence of the typical architectural pattern of growth of low-grade osteosarcoma and higher nuclear atypia), tumor maps were examined and the percentage of high-grade (grade 3) areas scored (Fig. 2a–c).

After definitive surgical treatment, the use of postoperative chemotherapy was mainly based on clinical experience, as no specific guidelines were available. For patients younger than 40 years, the chemotherapy regimen was based on the protocol adopted at the time for non metastatic osteosarcoma. The remaining patients received a chemotherapy regimen without methotrexate and usually including doxorubicin, cisplatin and ifosfamide. All patients, regardless of the use of adjuvant chemotherapy, were followed up every 2–3 months in the first 2–3 years and then every 4–6 months. Survival analysis was performed according to Kaplan–Meier. All analyses were performed using Stata/SE statistical software (version 10.0; StataCorp LT, College Station, TX, USA).

## Results

Of the 132 patients with a diagnosis of low-grade (grade 1 and 2) osteosarcoma at surgical biopsy, 33 patients were considered eligible for the study. The clinical and pathological features are summarized in Table 1. The median age was 37 years (range 13–58 years) with a slight prevalence of females. Most of the tumours were located in the extremities. The majority of the patients (26 cases, 79 %) had a diagnosis of fibroblastic osteosarcoma (of these, 4 cases had the fibrous dysplasia-like variant), followed by osteoblastic osteosarcoma (5 cases, 15 %) and chondroblastic osteosarcoma (2 cases, 6 %). All patients were surgically treated with wide surgical margins. Twenty-two (67 %) patients received adjuvant chemotherapy.

**Table 1:** The clinical and pathological features of 33 cases of low-grade central osteosarcoma with areas of high-grade (grade 3)

Case	Age/sex	Site	% high-grade (grade 3) areas	Histotype	Adjuvant chemotherapy	Local recurrence (months after surgery)	Metastases (months after surgery)	Follow-up (months after surgery)
1	56/F	Distal femur	2	Osteoblastic	No			NED (55)
2	39/M	Proximal humerus	4	Fibroblastic (fibrous dysplasia-like)	Yes		Lung (13)	DOD (39)
3	19/M	Distal femur	4	Osteoblastic	No			NED (157)
4	34/F	Proximal humerus	4	Fibroblastic	No			NED (120)
5	35/F	Distal femur	4	Fibroblastic	Yes			NED (225)
6	44/M	Proximal humerus	4	Fibroblastic	Yes			NED (56)
7	23/M	Distal femur	4	Fibroblastic	Yes			NED (69)
8	25/F	Distal femur	4	Fibroblastic	No	Yes (53)		NED (284)
9	13/F	Clavicle	9	Fibroblastic (fibrous dysplasia-like)	Yes			NED (103)
10	22/F	Proximal femur	9	Fibroblastic	Yes			NED (132)
11	32/F	Proximal humerus	9	Fibroblastic	No			NED (109)
12	58/F	Proximal femur	11	Fibroblastic	No			DOC (136)
13	27/M	Proximal tibia	16	Fibroblastic	No			NED (231)
14	45/M	Distal tibia	16	Fibroblastic	Yes			NED (45)
15	24/F	Distal femur	16	Osteoblastic	Yes			NED (45)
16	41/M	Proximal tibia	16	Osteoblastic	No			NED (89)
17	55/F	Pelvis	31	Osteoblastic	Yes			NED (33)
18	28/M	Proximal humerus	31	Fibroblastic (fibrous dysplasia-like)	No		Bone thoracic vertebra (51)	NED (57)
19	50/M	Distal femur	35	Fibroblastic	No			NED (262)
20	21/M	Distal femur	40	Fibroblastic	Yes			NED (151)
21	42/M	Proximal femur	51	Fibroblastic	Yes	Yes (21)	Lung (46; 65)	DOD (108)
22	38/F	proximal humerus	51	Fibroblastic (fibrous dysplasia-like)	Yes			NED (134)
23	59/M	Proximal humerus	51	Fibroblastic	Yes		Lung (37; 85)	NED (137)
24	35/F	Distal ulna	51	Fibroblastic	Yes			NED (4)
25	25/M	Lumbar vertebra	51	Fibroblastic	Yes			NED (10)
26	24/M	Proximal humerus	51	Osteoblastic	Yes			NED (65)
27	40/M	Proximal tibia	81	Fibroblastic	Yes		Lung (10)	DOD (32)
28	53/F	Proximal tibia	81	Fibroblastic	Yes	Yes (2)		DOD (5)
29	46/F	Distal femur	81	Fibroblastic	Yes		Lung (14;38;50)	NED (132)
30	49/F	Distal femur	81	Chondroblastic	Yes			NED (164)
31	42/F	Pelvis	81	Fibroblastic	Yes			NED (27)
32	20/F	Proximal femur	81	Chondroblastic	No			NED (250)
33	48/F	Distal femur	81	Fibroblastic	Yes			NED (322)

Follow-up data were available for all patients with a median observation time of 115 months (range 4–322 months). Twenty (61 %) patients showed areas of high-grade (grade 3) osteosarcoma accounting for less than 50 % of the tumor, whereas 13 (39 %) patients showed a high-grade (grade 3) component in the majority of the tumor (Table 1).

### Low-grade osteosarcoma with high-grade foci (grade 3 in less than 50 % of the tumor: 20 patients)

None of the patients belonging to this group showed metastatic lesions at the staging workup. Adjuvant chemotherapy was not given to 9 (45 %) patients. One patient (case 8) showed a local recurrence in the soft tissue 53 months after surgical resection. Recurrence was surgically removed and morphologically it was associated with progression to a higher grade (grade 4, fibroblastic and osteoblastic osteosarcoma). Therefore the patient received adjuvant chemotherapy and at the last follow-up (232 months after the surgical bone resection) is alive without disease. Another patient (case 18) developed a single bone metastasis in the thoracic vertebra, which was surgically removed. Morphologically the vertebral lesion showed the same features as the primitive osteosarcoma (grade 2 fibroblastic osteosarcoma, fibrous dysplasia-like variant). The patient started adjuvant chemotherapy and at the last follow-up (57 months after the surgical bone resection) is alive without disease. Another patient of this group (case 12) died of unrelated causes. The remaining patients are disease-free at last follow-up. Adjuvant chemotherapy was given to 11 (55 %) patients. One patient (case 2) developed multiple lung metastases 13 months after surgery. Histologically, the lung metastases were diagnosed as grade 4 osteoblastic osteosarcoma. After surgical resection of the lung metastases the patient underwent second line chemotherapy and died of disease 39 months after the first diagnosis.

Overall 18 (90 %) of the 20 patients with an area of high-grade (grade 3) osteosarcoma accounting for less than 50 % of the tumor were alive without disease, none of the patients who did not receive adjuvant chemotherapy died of disease. The median observation time was 109 months (from 29 to 284 months). The probability of evidence-free survival and overall survival at 5 years was 88 % (95 % CI 72–100 %) and 95 % (95 % CI 84–100 %), respectively.

### Low-grade osteosarcoma with high-grade foci (grade 3) in more than 50 % of the tumor (13 patients)

Twelve patients received adjuvant chemotherapy. Of these, two patients had recurrent disease in the soft tissues (cases 21, 28) after 2 and 21 months of follow-up, respectively, and one of them (case 28) died of disease 5 months after the surgical resection. Four patients developed lung metastases (cases 21, 23, 27, 29) after a mean follow-up of 27 months (range 10–46 months). Three of these 4 patients (cases 21, 23, 29) developed other multiple lung metastases following the first lung metastasectomies. Both the two recurrences and lung metastases were histologically associated with progression to a higher grade (grade 4) osteosarcoma.

Two of 4 patients with lung metastases (cases 21, 27) died of disease after 108 and 32 months, respectively. The patient (case 32) who did not receive chemotherapy is alive without disease 232 months after complete surgical remission.

Overall 10 (77 %) of the 13 patients with areas of high-grade (grade 3) osteosarcoma accounting for more than 50 % of the tumor were alive without disease, 5 (38.5 %) patients experienced recurrent disease, with distant metastases in 4 and local recurrence in only one patient. The median observation time was 120 months (from 4 to 322 months). The probability of evidence-free survival and overall survival at 5 years was 68 % (95 % CI 42–94 %) and 83 % (95 % CI 61–100 %), respectively.

## Discussion

The grading of osteosarcoma is based on morphologic observation of a set of parameters that have proved relatively reproducible [1, 2, 4]. Some discrepancies are unavoidable when comparing biopsy material with the corresponding surgical specimen, in which variable amounts of higher grade malignancy can be seen. It is our experience that areas of high-grade (grade 3) osteosarcoma can be found in patients with a bioptic diagnosis of low-grade (grade 1–2) osteosarcoma, even when the biopsy was obtained from the site showing the greatest aggressiveness on imaging studies [6–8, 17, 18].

Interestingly, the group of patients forming the study population exhibit clinical features that differ from those observed in patients with conventional high-grade osteosarcoma. The age of our patients was higher than the age reported for classic osteosarcoma, with most of the patients being adults [1, 2, 4]. Another observed difference was the histologic subtype. It is well known that osteoblastic osteosarcoma is the most frequent subtype [1, 2, 4], whereas most low-grade osteosarcomas with high-grade areas were fibroblastic osteosarcoma.

High-grade dedifferentiation or progression to a higher grade in low-grade central osteosarcoma represents an exceedingly rare event, which can be seen in the primary tumor, but more commonly in recurrences [7–12, 19]. Our series reports a 25 % rate of progression to high-grade (grade 3) osteosarcoma (33/132 cases), which to some extent overlaps with the range of 10–36 % of cases reported in the literature [3–5, 7–12]. Previous reports have documented the association between inadequate surgical resection, local recurrence, and morphologic progression to higher grade, which was associated with a poor prognosis [7, 8, 19]. While surgery alone is deemed adequate for low-grade central osteosarcoma, even in the absence of published guidelines, the addition of chemotherapy is being suggested as an option for those patients featuring areas of histologic progression (also defined by some authors as dedifferentiation) [7–15, 19].

This study shows that the presence of areas of high-grade (grade 3) progression in patients with low-grade osteosarcoma does not by itself imply biological systemic aggressiveness. Furthermore we have shown a correlation between the percentage of high-grade (grade 3) areas and risk of metastatic spread. In our retrospective analysis, those patients with low-grade osteosarcoma with a high-grade (grade 3) component of less than 50 % of the tumor did in fact have a very high probability of survival regardless of the use of adjuvant chemotherapy. By contrast, the clinical behaviour of tumors featuring a high-grade (grade 3) osteosarcoma component greater than 50 % overlaps with that reported for conventional high-grade osteosarcoma [1, 2, 4, 17, 18].

In surgical pathology it is broadly accepted that the grade of any tumor is based on the highest grade observed in the surgical specimen [20]. Our study seems to suggest that this general rule cannot be applied systematically to all osteosarcomas.

The standard treatment of high-grade (grade 3–4) osteosarcoma is surgical removal of the tumor and adjuvant (or neo-adjuvant) chemotherapy [3, 13–15]. A pathology report describing areas of high-grade (grade 3–4) osteosarcoma would currently represent the rationale for the use of chemotherapy [3, 13–15].

## Conclusion

Our data indicate that not all patients with a diagnosis of osteosarcoma featuring a high-grade (grade 3) component would benefit from systemic treatment. In particular, in patients with small foci with high-grade (grade 3) progression or in whom the high-grade (grade 3) component is less than 50 % of the resected specimen at tumour map examination, high survival rates can only be achieved by means of complete surgical resection.
